# Postpartum maternal bonding problems relate to aberrant neural processing of infant emotions: Results of an adapted fMRI emotional GoNoGo task

**DOI:** 10.1038/s41398-026-04231-y

**Published:** 2026-07-06

**Authors:** Monika Eckstein, Marlene Krauch, Ines Brenner, Beate Ditzen, Anna-Lena Zietlow

**Affiliations:** 1 Institute of Medical Psychology, University Hospital Heidelberg, Heidelberg, Germany; 2 Clinical Child and Adolescent Psychology, Faculty of Psychology, Dresden University of Technology, Dresden, Germany; 3 Clinical Biopsychology and Psychotherapy, Department of Psychology, University of Zurich, Zurich, Switzerland

**Keywords:** Depression, Human behaviour, Learning and memory

## Abstract

Maternal bonding refers to the unique emotional connection between a mother and her baby that gradually develops during the peripartum period. However, 3–24% of women report bonding problems (BP), often accompanied by constraints for the mother-infant relationship, but not always depression, with consequences for child development. Our present study investigates the neural and behavioral patterns that underlie the processing of emotional infant stimuli at 3 months postpartum, with an additional exploratory perspective over the 1st year postpartum parallel to a neurofeedback intervention that took place between 3 and 6 months postpartum. Mothers with and without BP (*N* = 45) completed a newly developed Emotional Infant GoNoGo Task during fMRI scanning at 3, 6 and 12 months postpartum. Our results show that response inhibition towards emotional infant faces elicits stronger results than towards adult faces in all mothers, on a neural as well as on a behavioral level. The neural responses to emotional infant faces as compared to neutral faces are increased at 3 months postpartum in limbic structures such as the anterior cingulate and insula, as well as nucleus caudatus, indicating altered emotion processing in mothers with postpartum bonding problems. Explorative and preliminary analyses for 6 and 12 months postpartum found differences in neural and behavioral reactions between BP and healthy controls increase at 6 months and decrease again at 12 months, may point to an experience-based adaptive process of infant emotion processing in mothers with BP during the first year postpartum. Clinical prevention and intervention strategies for mothers with postpartum BP should therefore focus on emotion processing and regulation capacities, particularly during the first months postpartum.

## Introduction

Maternal bonding refers to the unique emotional bond between a mother and her infant that already begins during pregnancy and continues to develop during the postpartum period [1, 2]. Maternal bonding is evident behaviorally through cuddling, smiling at the infant, nurturing behaviors, and a high sensitivity to the infant’s needs [3]. On a cognitive level, it involves the mother’s thoughts about the infant and his/ her needs [4]. On a neurobiological level, bonding is associated with alterations in structural and functional brain changes [5–7] and hormonal regulation [8]. Strong maternal bonding can substantially improve infant development, influencing emotional and cognitive trajectories (for review see Le Bas, Youssef, [9]). Maternal bonding is related to neural sensitivity to infant-related stimuli, for example infant facial expressions [10, 11]. The correct interpretation of infants’ facial cues is crucial because, compared to adults and older children, infants have limited social skills and primarily communicate through facial expressions, body language, and vocalisations (see Bowlby, [12, 13]). Therefore, correct and prompt maternal perception and processing of the infant’s emotional cues is essential for appropriate maternal responses to the infant’s needs.

Research shows that healthy mothers demonstrate stronger and quicker neural responses to infant stimuli compared to non-mothers in crucial emotional processing regions such as the amygdala, insula, and orbitofrontal cortex. This response is particularly pronounced when interacting with their own infants [14, 15]. This increased responsiveness to infant cues can be partly explained by neurobiological changes during transition to parenthood [6, 7, 16, 17] and also includes an altered response to infant stress cues [18]. Functional and structural changes are observed in core limbic and cortical socio-cognitive networks that are unique to parents and related to caregiving behavior [17, 19, 20]. It is assumed that these alterations endure (see [21] for review).

Healthy mothers with a close maternal-infant bond show specific patterns of neural activity when responding to negative emotional infant stimuli. In particular, regions such as the amygdala, anterior cingulate cortex (ACC), ventral prefrontal cortex (PFC) and insular cortex are associated with emotion processing and regulation of negative emotional infant stimuli [15, 22]. On a neural level, a child’s face triggers stronger activation in the fusiform gyri, the middle occipital gyri, the superior temporal gyri, the supplementary motor area, the pre- and postcentral gyri, as well as in the amygdala, when compared to an adult’s face (for review see [23]). Interestingly, these effects in mothers are often lateralized to the right hemisphere [23]. However, previous studies differ in the methods they use (own or unfamiliar child, visual or acoustic stimuli, infant or adult facial stimuli). E.g., Rupp et al. [24] investigated mothers compared to non-mothers when exposed to negative emotional infant images and report reduced activation in the right amygdala and reduced subjective negative arousal in mothers. Similarly, in an Emotional GoNoGo Task, mothers showed increased NoGo P3 amplitudes compared to non-mothers, underlining differences in emotional regulatory mechanisms between mothers and non-mothers [18].

Research in this area underscores the significance of emotion regulation in tempering over-reactivity to infant’s crying, a factor closely associated with maternal mental health [25, 26]. Emotion regulation can be considered a higher-order process that follows the initial steps of direct emotion processing (i.e., attention to and perception of information eliciting emotional arousal) and the subsequent reaction to these stimuli. Inhibition of a direct response in order to avoid negative consequences requires medium-level brain circuitries, such as the limbic system including the cingulate cortex, but not cognitive executive function [27]. Proficiency in utilizing multiple strategies to regulate emotions may serve to prevent high parental stress and mental illness [28]. Specifically mothers’ sensitivity to their infants’ facial expressions is correlated with activation in brain regions associated with emotion regulation, particularly behavioral inhibition [18], while emotion regulation in a broader sense seems impaired in postpartum mental disorders such as depression and anxiety [29, 30].

Maternal characteristics and health are thought to affect the neural processing of infant stimuli, e.g. maternal sensitivity influences neural activation in response to infant stimuli [31–35]. Previous research suggests, that mothers who are more sensitive in the interaction with their child, show greater activation in the right frontal pole and inferior frontal gyrus when listening to their own baby crying as compared to an unfamiliar infant 18 months postpartum. Mothers interacting less sensitive showed higher activation in the left insula and temporal pole [35]. Regarding postpartum depression, several studies suggest a reduced processing of infant faces [36–38]. For instance, Moses-Kolko et al. [38], investigated neural reactions to negative facial expressions using fMRI in both, mothers diagnosed with postpartum depression and a control group of healthy individuals. The results suggest that reduced activity in the dorsomedial PFC and reduced effective connectivity between the dorsomedial PFC and the amygdala in response to negative emotional faces may reveal a crucial neural mechanism or consequence of postpartum depression.

The presence of a clinical maternal BP may be another possible influential factor for impaired infant emotion processing. A notable portion of women, specifically 3 to 24% in community samples [3, 39–45] face challenges in forming a good emotional bond with their offspring. While BP can indeed manifest in healthy mothers, it occurs more frequently in the context of perinatal mental illness such as perinatal depression, anxiety, or post-traumatic stress disorder [42, 46, 47]. For mothers with perinatal depression, the prevalence rates of BP range from 17–29% [48]. BP show in various dimensions, including emotional disconnection, and feelings of rejection or frustration towards the infant [43, 49, 50]. As maternal caregiving behavior may be restricted in mothers with BP [51] this is probably paralleled by neural alterations. Accordingly, mothers who reported poorer postpartal bonding have been reported to process stressed infant faces slower in late pregnancy [52]. In addition, faster processing of infant faces compared to adult faces in an EEG task during pregnancy has been reported to be associated with higher maternal bonding quality [11]. Maternal depression and bonding problems are even associated with deficits in child development [53, 54].

Behavioral inhibition towards social emotional stimuli can be assessed using a GoNoGo paradigm that requires the individual to correctly identify emotions and flexibly change his/her reaction to them. As task performance is often impaired in persons burdened by mental health issues [55, 56], a non-social GoNoGo Task is an established fMRI research paradigm. The extension to adult facial stimuli is especially utilized in research on disorders that are characterised by social deficits (e.g. depression [57]).

However, maternal bonding problems in particular have so far not been systematically investigated in terms of behavioral and neural responses to emotional child-related stimuli. Yet, understanding the processing of emotional infant stimuli and subsequent maternal emotion regulation may help to explain the parenting difficulties of mothers with BP. Based on the state of research summarized above, we expect behavioral and neural deficits processing infant and adult faces within a GoNoGo Task in mothers with BP compared to a control group.

The present study is part of a larger project including an fMRI-neurofeedback intervention [58]. Details and results of the intervention part are submitted for publication elsewhere (see preprint at [59] Zietlow et al. 10.21203/rs.3.rs-8166275/v1). Here we present results of an adapted version of the Emotional GoNoGo Task for fMRI using infant stimuli. We focus on investigating the neural mechanisms underlying inhibitory responses to emotional infant faces compared to adult faces in women at 3 months postpartum. We analyze behavioral outcomes associated with BP, focusing on reaction times and error rates during inhibition of responses to emotional baby faces compared to emotional adult faces in order to describe the neurobehavioral response patterns in women with BP to emotional infant stimuli during the postpartum period (and disentangle them from depressive symptoms). Based on the state of research summarized above, we expect to detect group differences at 3 months postpartum in behavior and neural key nodes related to parental emotion regulation such as the ACC. Additional exploratory analyses are performed for 6 and 12 months postpartum, to investigate group differences during the first year postpartum.

## Methods

**Fig. 1:**
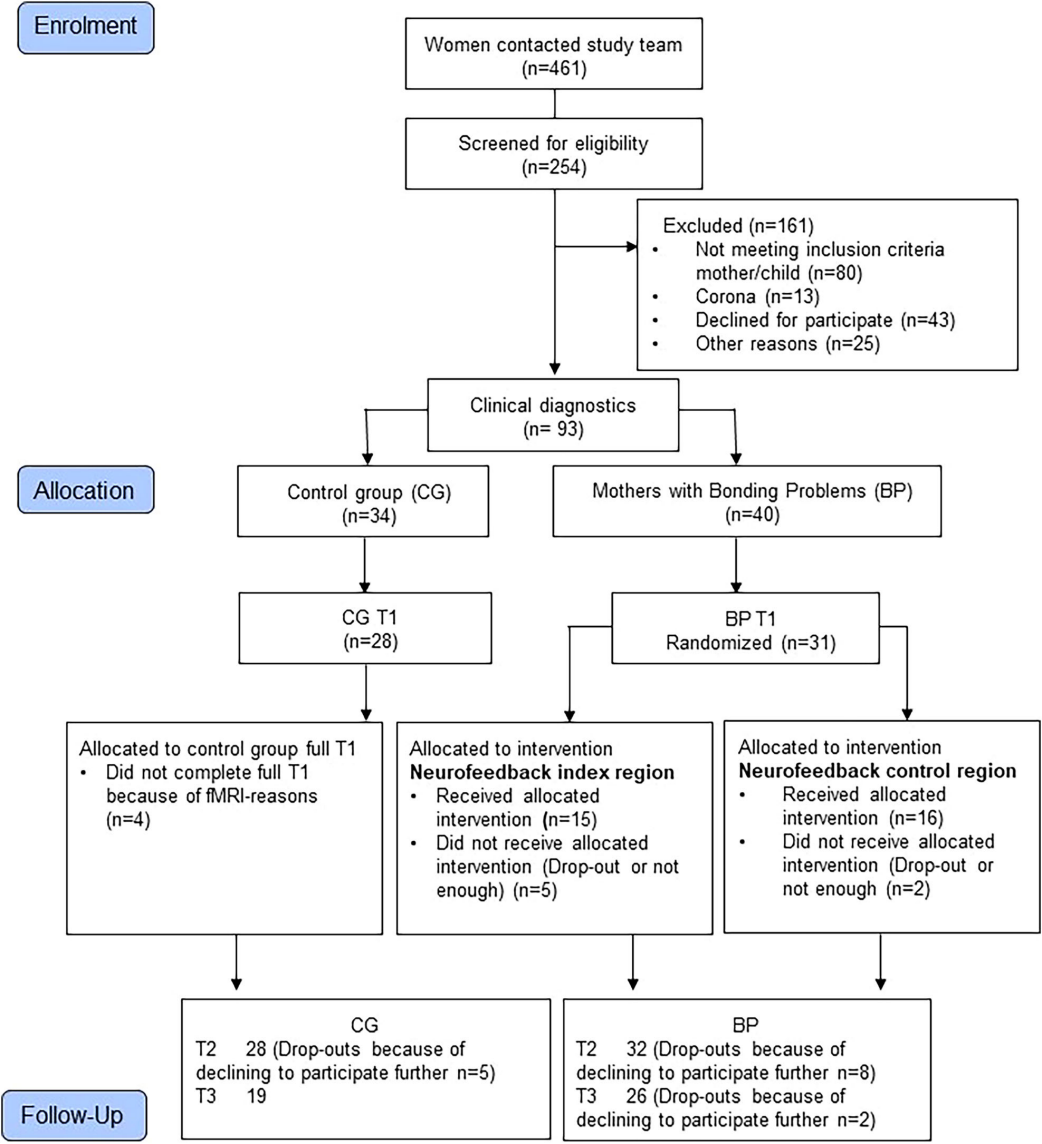
Study Participant Flow Chart.

A total of *N* = 64 participants were recruited as part of a larger study on postpartum bonding disorders (for details see [58], approval was given by Ethics Committee of the Medical Faculty Heidelberg, S-450/2017). Recruitment was done mainly via data of registration offices. Following initial contact of 461 mothers, a phone-screening interview with 254 was administered 4 to 10 weeks postpartum to address preliminary exclusion criteria (see Fig. 1). Exclusion criteria were severe psychiatric conditions that needed treatment, substance abuse, pre-term birth, multiple birth, severe health issues of the baby and MRI contraindications.

Subsequently, based on the results of the mother’s bonding interview conducted with 93 women based on the proposed criteria at T1, mothers were assigned to either the intervention (BP, n = 40) or control group (CG, N = 34). All participants were subjected also to detailed psychodiagnostics according to DSM-5 over the course of the study. Participants performed three task-based fMRI sessions over the first year postpartum: with approx.3 months (T1), 6 months (T2) and 12 months (T3) postpartum. During these sessions, they first performed a monetary reward task, subsequently the Infant Emotional GoNoGo Task and a passive viewing task of pictures of their own child and partner. The BP group received a neurofeedback intervention between T1 and T2. Results on the intervention effects and other tasks are currently under review (for preprint see 59, 10.21203/rs.3.rs-8166275/v1). Results not central to the present research question will be published separately.

### fMRI task: infant emotional GoNoGo task

**Fig. 2:**
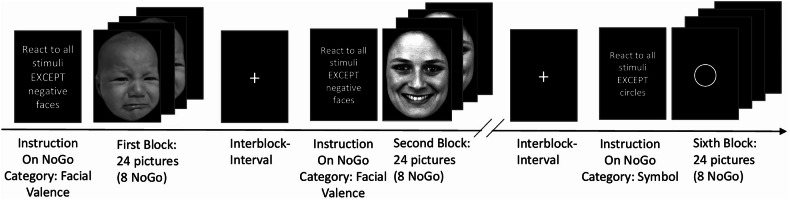
fMRI paradigm: the Infant Emotional GoNoGo Task comprising six blocks in randomized order.

Using a specifically adapted GoNoGo paradigm, participants were presented with positive, negative and neutral expressions of pictures of unknown babies, aged approx. 4–10 months [60], and unknown adults, as well as non-social stimuli, see Fig. 2.

In two blocks, the participants received instructions to respond by pressing a button as fast as possible (Go trials) to all facial expressions except (NoGo trials) the negative. In two other blocks, they were instructed to respond as fast as possible to all except the positive stimuli. Details on the task are in the Supplemental methods.

### Self reports

The assessment of bonding problems was conducted using an interview based on the criteria outlined by Brockington [48]. These criteria categorize bonding problems into delay/loss of bonding, pathological anger and rejection. Maternal depression was screened using the Edinburgh Postnatal Depression Scale (EPDS) [61]. fMRI data acquisition is described in the supplemental material.

### Analyses

Behavioral data were analysed using IBM SPSS Statistics (version 31). We focused on reaction times (go trials) and on error rates of inhibition (nogo trials). Data for T1 were analysed using paired-samples t-tests and MANOVAs for between-group comparisons. In an exploratory approach, mixed ANOVAs were conducted to compare reaction times and error rates across T1, T2 and T3.

For fMRI data, using SPM12 (Wellcome Center for Human Neuroimaging, London, UK) we specified an event-related model with 3 sessions (T1, T2, T3) and the 12 task conditions with the onsets of symbols, adult positive faces, adult negative faces, baby positive faces and baby negative faces, both as Go and NoGo conditions, and neutral adult and neutral baby faces as Go condition. The estimated model calculated contrasts for the relevant conditions such as T1 [emotional baby face > neutral baby face] and Mean [[baby face NoGo > baby face Go] > [adult face NoGo > adult face Go]]. On the second level, participant-specific contrast maps from the first level analyses were compared between mothers with BP and healthy controls with two-sample t-tests and EPDS scores were tested as covariates. See further details in the supplementary material.

A post hoc sensitivity analysis in G*Power [62], for repeated measures ANOVA, α = 0.05, power = 0.80) indicated that with two groups of N = 31 the smallest detectable effect size was f = 0.29 which corresponds to approx. η^2^ = 0.08. While small samples may increase uncertainty in effect size estimation, the sensitivity analysis suggests that the study was capable of detecting medium size effects only.

## Results

### Sample characteristics

*N* = 64 started in the overall project after giving written informant consent. Due to the Covid-19 pandemic, several fMRI sessions for T2 and T3 had to be cancelled, resulting in diverging sub-samples for some of the analyses, see Table 1 for description.

**Table 1:** Sample description for participants that completed at leastT1.

		Mothers with bonding problems (BP)			Control group (CG)			Difference
		N	Mean	SD	N	Mean	SD	
Depressiveness (EPDS)	T1	31	10.068	4.53	28	3.036	2.78	T(57) = 7.11, *p* = 0.021*
	T2	24	7.417	4.95	23	2.522	2.73	T(45) = 4.32, *p* < 0.001*
	T3	23	5.043	3.78	25	3.64	2.75	T(46) = 1.48, *p* = 0.073
Bonding Problems (PBQ)	T1	31	24.709	11.21	28	5.607	3.39	T(57) = 8.66, *p* < 0.001*
	T2	26	15.077	6.09	26	5.039	2.27	T(50) = 7.54, *p* < 0.001*
	T3	23	15.043	9.65	23	6.68	5.52	T(46) = 3.72, *p* < 0.001
Maternal age at delivery		30	32.93	3.54	28	31.39	3.45	T(56) = 1.67, *p* = 0.869

*PBQ* postpartum bonding questionnaire [3], * significant result.

*N* = 55 completed the first fMRI Session (T1), *N* = 31 mothers with BP, *N* = 28 healthy controls. Complete data of *N* = 45 persons for the three fMRI sessions at 3, 6 and 12 months was available that entered the analyses reported below. Of these 45 mothers, *N* = 19 built the healthy control group and *N* = 26 the group of mothers with BP. The BP group was further allocated to either an index neurofeedback intervention or a control neurofeedback intervention between 3 and 6 months. No neural or behavioural differences between these two intervention groups were observed for the present fMRI task. Therefore, data was pooled. The focus of interpretation is on the results of T1 that are not influenced by the intervention.

In the BP group, 12 mothers (36.36%) reported current depression, 5 mothers (15.15%) were diagnosed with a current anxiety disorder, and 9 mothers (27.27%) had depression with comorbid anxiety. For lifetime diagnoses, 2 mothers (6.06%) reported lifetime depression, and in each case, one mother had an anxiety disorder or depression with comorbid anxiety disorder. In the BP group, 3 mothers (9.09%) did neither meet criteria for any current nor lifetime mental disorder. Find details on correlations between BP and depression in the supplementary results.

## Behavioral results

### 3 months postpartum

#### Main effect of task

**Fig. 3:**
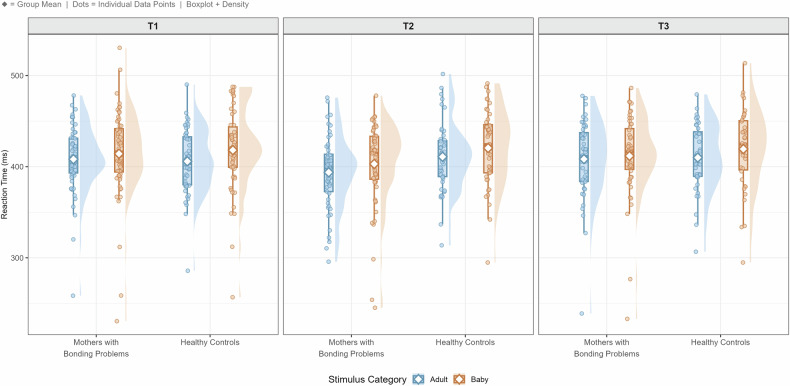
Response times in Go condition: Raincloud plot displaying the distribution of reaction times at T1, T2, T3. Boxes indicate the median and interquartile range (IQR); whiskers extend to 1.5 × IQR; dots represent individual observations.

At T1, in the emotional go-condition, the paired-samples t-test across all participants, mothers with BP and healthy controls, revealed no significant differences in reaction times for baby faces vs. adult faces t(62) = − 1.44, p = 0.156, see Fig. 3.

**Fig. 4:**
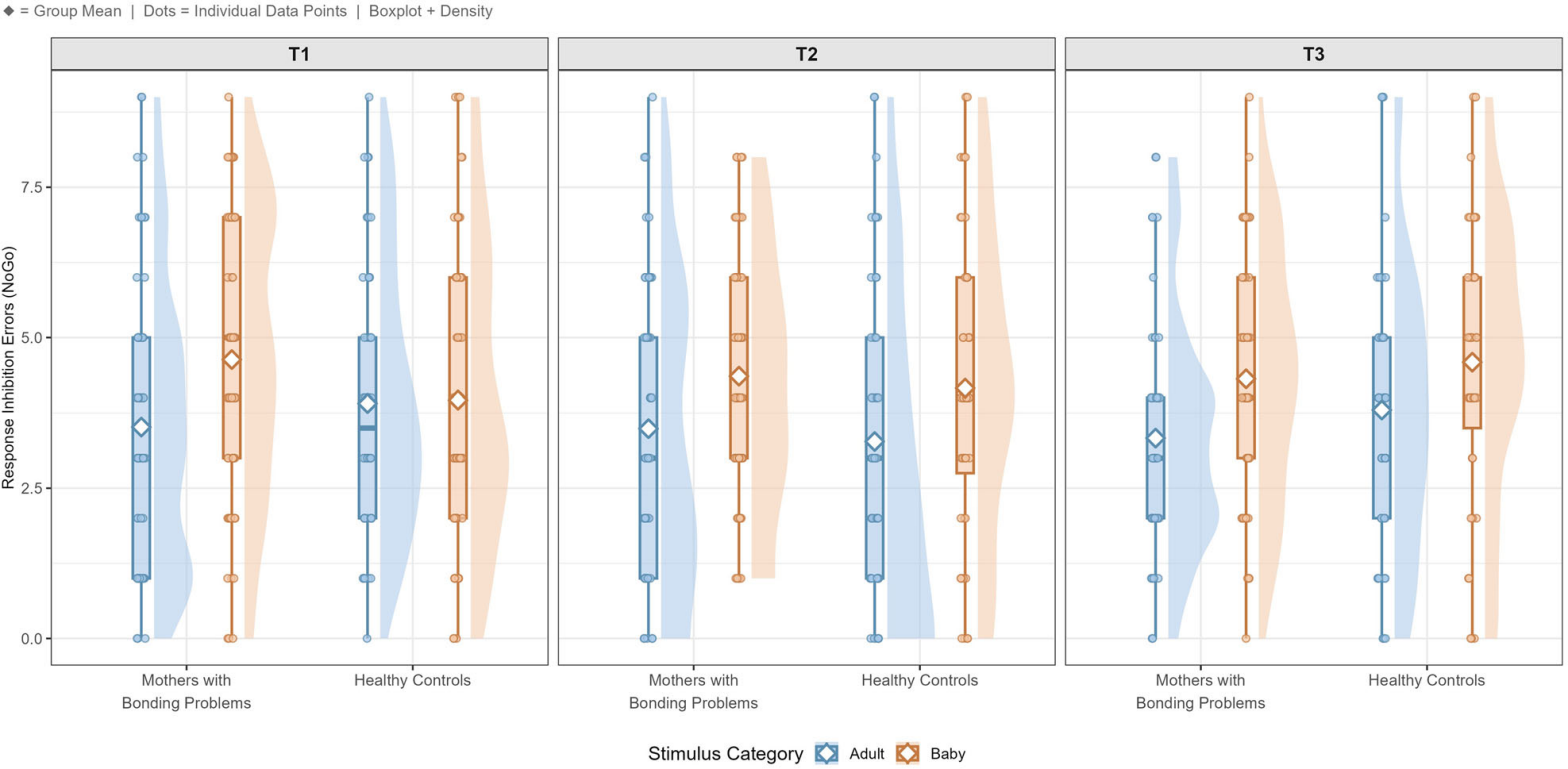
Accuracy in NoGo condition: Raincloud plot displaying the distribution of error rates at T1, T2, T3. Boxes indicate the median and interquartile range (IQR); whiskers extend to 1.5 × IQR; dots represent individual observations.

In the emotional nogo-condition, at T1, the paired-samples t-test across all participants revealed significant higher error rates of inhibition for baby faces (M = 4.39, SD = 2.20) than for adult faces (M = 3.70, SD = 1.64), t(62) = −3.25, p = 0.002, d = 0.41, see Fig. 4.

#### Group comparison

Regarding group differences between mothers with BP and healthy controls at T1, neither the MANOVA for reaction times, F(12,24) = 0.55, p = 0.859, partial η² = 0.216, nor the MANOVA for inhibition error rates, F(6,32) = 1.19, p = 0.336, partial η² = 0.183, were significant.

### 6 and 12 months postpartum

#### Main effect of task and group comparison

The exploratory mixed ANOVA with the within-subject factors time (T1, T2, T3) and faces (baby, adult) and the between-subject factor group (mothers with BP, control group) revealed a main effect of faces, F(1,36) = 8.55, p = 0.006, partial η² = 0.192, indicating an overall significant difference in reaction times between baby and adult faces across groups, with slower reactions to baby faces than to adult faces, see Fig. 3. No significant main effects of group or time and no significant interaction effects were observed (all *p* > 0.169), suggesting that the difference in reaction times between face types did not significantly change over time.

Similarly, the exploratory mixed ANOVA for inhibition error rates with the within-subject factors time (T1, T2, T3) and faces (baby, adult) and the between-subject factor group (mothers with BP, control group) revealed a main effect of faces, F(1,42) = 29.51, *p* < 0.001, partial η² = 0.413, indicating an overall significant difference in inhibition error rates between baby and adult faces, with higher error rates for baby faces, see Fig. 4. Additionally the main effect of time was significant, F(2,41) = 4.75, *p* = 0.014, partial η² = 0.188. Furthermore, the interaction time*group was significant, F(2,41) = 7.34, *p* = 0.002, partial η² = 0.264, indicating that the changes in inhibition error rates over time differed between the two groups. To decompose this interaction, simple main effects for group were analyzed using Sidak-adjusted pairwise comparisons. Results indicated no significant difference between groups at T1, *p* = 0.654. However, a group difference emerged at T2 at a trend level, *p* = 0.085, with the mothers with bonding problems showing lower error rates of inhibition compared to healthy controls. At T3, there were again no significant differences between groups, *p* = 0.637.

## fMRI results

All analyses were first conducted with EPDS scores as covariate, which did not reach any significance. Therefore, we report the parsimonious results without EPDS.

### Main effects of task

**Fig. 5:**
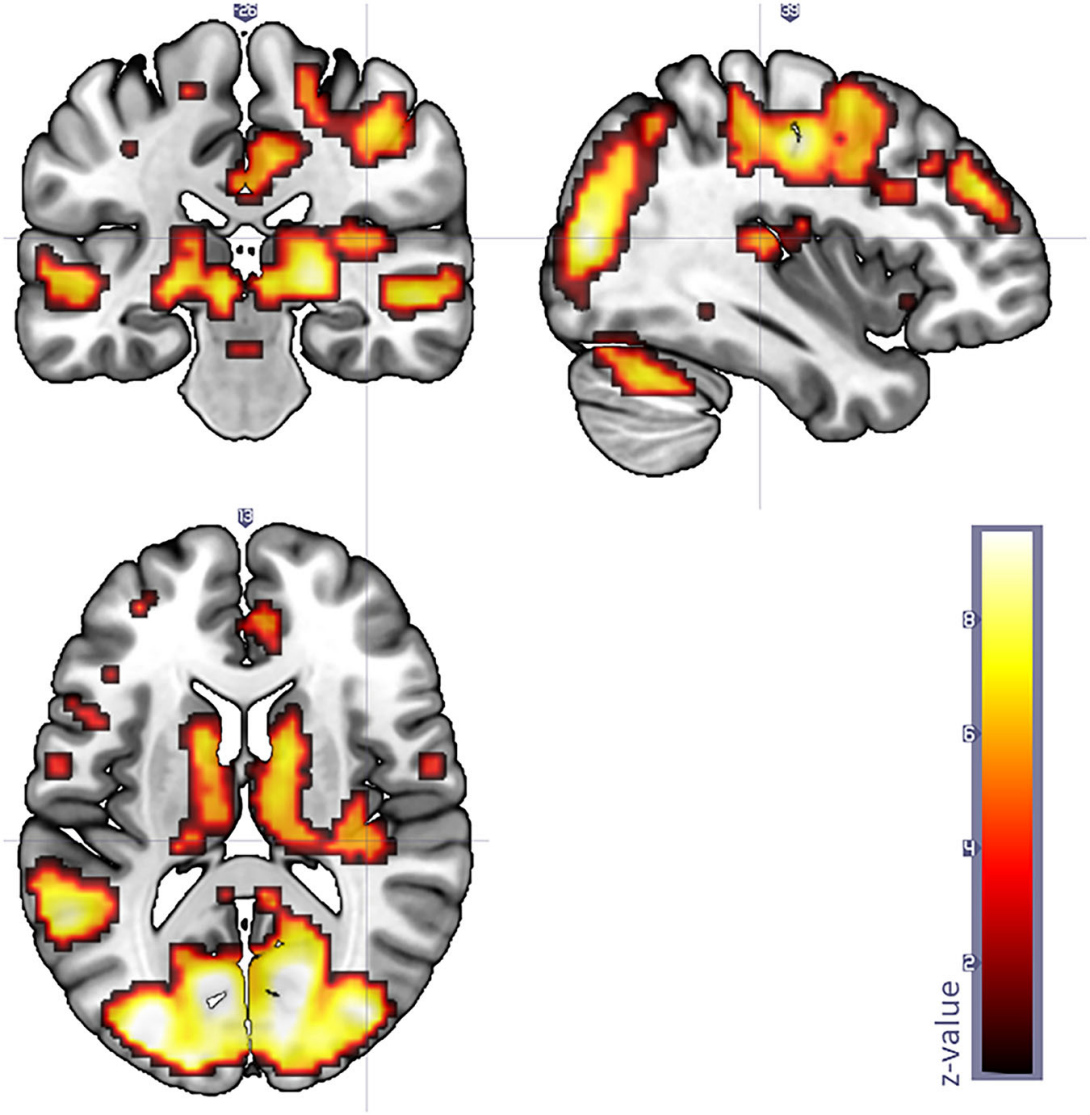
Functional activation shown in three orthogonal planes (axial, sagittal, and coronal). Neural activation of inhibition towards NoGo baby stimuli as compared to non-inhibition (=reaction, Go Condition) over all participants and assessments.

The Infant Emotional GoNoGo Task activated a broad network of emotion processing and emotion inhibition over all participants and time points as assessed by contrasting [baby faces NoGo > baby faces Go] irrespectively of emotional valence. Among others, significant activations were found in anterior and middle cingulate cortex, inferior frontal gyrus, temporal gyrus and insula, see Fig. 5 and Table 2.

**Table 2:** Activations pattern for the baby NoGo condition as compared to the Go condition.

cluster	cluster size	peak	peak	peak	NMI coordinates (mm)			hemis-phere	region
p(FWE-corr)	equivk	p(FWE-corr)	T	equivZ	x	y	z		
0.00	10231.00	0.00	15.81	65535.00	−15	−91	−4	L	calcarine cortex
		0.00	15.26	65535.00	18	−91	−1	R	calcarine cortex
		0.00	15.21	65535.00	−9	−85	2	L	calcarine cortex
0.00	758.00	0.00	12.58	65535.00	−42	2	47	L	precentral gyrus & middle frontal gyrus
		0.00	11.14	7.56	−45	−4	35	L	precentral & postcentral gyrus & middle frontal gyrus
		0.00	8.62	6.51	−48	14	23	L	inferior frontal. middle frontal and precentral gyrus
0.00	673.00	0.00	9.74	7.01	−3	8	62	L	supplementary motor cortex and superior frontal gyrus
		0.00	7.23	5.79	9	14	56	R	supplementary motor cortex and superior frontal gyrus
		0.00	7.18	5.77	6	26	35	R	supplementary motor cortex anterior and middle cingulate and superior frontal gyrus
0.00	147.00	0.00	8.29	6.35	54	−25	−7	R	middle and superior temporal gyrus
		0.00	7.33	5.85	63	−28	−4	R	middle and superior temporal gyrus
0.00	43.00	0.00	6.94	5.63	54	−46	26	R	angular and supramarginal gyrus
0.00	42.00	0.00	6.82	5.57	−30	35	35	L	middle and superior frontal gyrus
0.00	15.00	0.00	6.65	5.47	−42	44	20	L	middle frontal gyrus
0.00	49.00	0.00	6.56	5.42	54	−4	−10	R	superior and middle temporal gyrus
		0.02	5.49	4.74	60	5	−4	R	superior temporal gyrus and temporal pole
0.00	22.00	0.00	6.11	5.14	−54	−7	−10	L	superior and middle temporal gyrus
0.00	17.00	0.01	6.01	5.08	9	−13	53	R	supplementary motor area and precentral gyrus
0.01	6.00	0.01	5.92	5.02	0	−37	−31		brain stem
0.02	2.00	0.01	5.64	4.84	−24	8	−13	L	putamen and anterior insula
0.00	13.00	0.02	5.58	4.80	−18	−37	59	L	postcentral gyrus and superior temporal lobule
0.01	3.00	0.02	5.57	4.79	−30	50	11	L	middle and superior frontal gyrus
0.01	5.00	0.02	5.56	4.78	54	26	26	R	middle and inferior frontal gyrus
0.01	4.00	0.02	5.54	4.77	54	−49	−10	R	inferior and middle frontal gyrus
0.01	3.00	0.03	5.43	4.70	54	14	−7	R	temporal pole and frontal operculum
0.01	3.00	0.03	5.42	4.69	57	23	20	R	inferior and middle frontal gyrus
0.01	4.00	0.03	5.41	4.68	0	−25	−22		brain stem
0.01	3.00	0.03	5.36	4.65	−18	−25	59	L	precentral and postcentral gyrus
0.03	1.00	0.03	5.35	4.64	36	20	−7	R	anterior insula and inferior frontal gyrus
0.03	1.00	0.04	5.30	4.61	9	−4	47	R	supplementary motor area and middle cingulate cortex
0.03	1.00	0.04	5.28	4.59	−9	−10	38	L	middle cingulate cortex and supplementary motor area
0.02	2.00	0.04	5.23	4.56	54	−43	41	L	supramarginal and angular gyrus

**Fig. 6:**
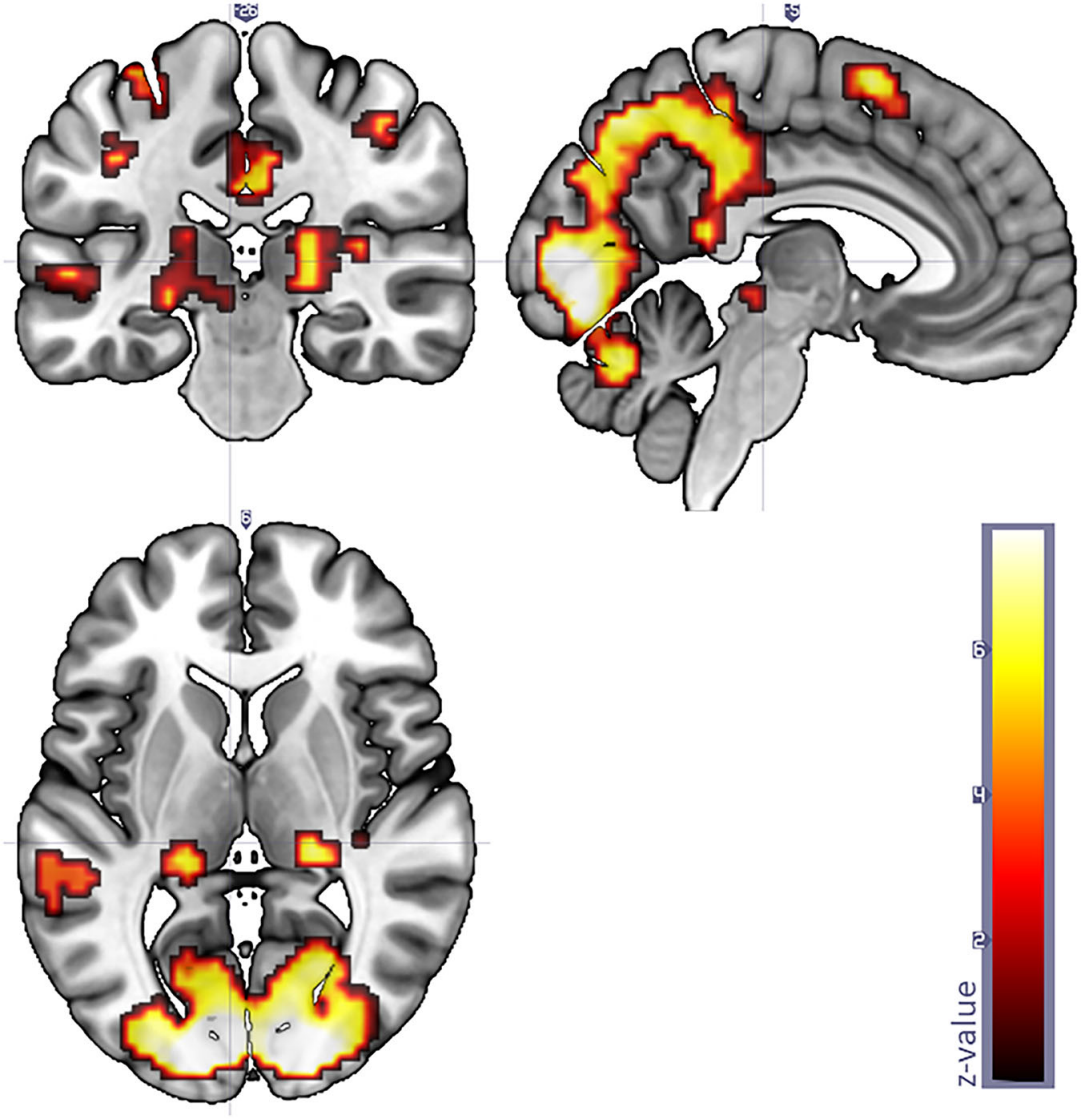
Functional neural activation of [inhibition towards baby stimuli as compared to non-inhibition] as compared to [inhibition towards adult stimuli as compared to non-inhibition] over all participants and assessments.

Comparing reactions toward adult faces with reactions towards baby faces [baby faces NoGo > baby faces Go] > [adult faces NoGo > adult faces Go] resulted in even stronger activation patterns for the baby stimuli in posterior cingulate, hippocampus and other key nodes for emotion processing, see Fig. 6 and Table 3.

**Table 3:** Activation pattern for the baby NoGo condition as compared to the Go condition.

cluster	cluster size	peak	peak	peak	NMI coordinates (mm)			hemis phere	region
p(FWE-corr)	equivk	p(FWE-corr)	T	equivZ	x	y	z		
0.00	6415.00	0.00	12.48	65535.00	−12	−88	−7	L	lingual gyrus and calcarine cortex
		0.00	11.87	65535.00	−12	−91	2	L	calcarine cortex and occipital pole
		0.00	11.54	7.82	6	−85	−7	R	lingual gyrus and calcarine cortex
0.00	42.00	0.00	7.01	5.74	−6	8	62	L	supplementary motor cortex and superior frontal gyrus
0.00	77.00	0.00	6.97	5.71	−24	−31	−1	L	thalamus and hippocampus
		0.03	5.30	4.65	−9	−28	−4	L	brain stem
0.00	56.00	0.00	6.78	5.60	21	−28	5	R	thalamus and hippocampus
0.00	268.00	0.00	6.58	5.48	42	−7	32	R	precentral and postcentral gyrus
		0.00	6.29	5.30	45	−10	44	R	precentral and postcentral gyrus
		0.00	5.87	5.03	63	−1	23	R	precentral and postcentral gyrus
0.00	31.00	0.00	6.19	5.24	−57	−52	17	L	superior and middle temporal gyrus
0.00	16.00	0.00	6.07	5.16	−12	17	−1	L	caudate, accumbens and putamen
0.00	20.00	0.01	5.85	5.02	−27	32	38	L	middle and superior frontal gyrus
0.01	8.00	0.01	5.66	4.90	33	11	53	R	middle and superior frontal gyrus
0.01	8.00	0.02	5.46	4.76	33	−25	11	R	temporal gyrus and posterior insula
0.00	11.00	0.02	5.43	4.73	24	26	41	R	middle and superior frontal gyrus
		0.04	5.11	4.51	27	32	35	R	middle and superior frontal gyrus
0.00	16.00	0.02	5.42	4.73	−30	−25	65	L	precentral and postcentral gyrus
0.01	6.00	0.02	5.40	4.72	30	41	35	R	middle and superior frontal gyrus
0.02	3.00	0.03	5.28	4.63	12	−34	59	R	precentral and postcentral gyrus and precuneus
0.02	3.00	0.03	5.26	4.62	36	−34	62	R	postcentral gyrus and superior parietal lobule
0.03	1.00	0.03	5.19	4.57	−9	−34	62	L	precentral and postcentral gyrus
0.02	2.00	0.03	5.18	4.56	9	−13	56	R	supplementary motor cortex and prefrontal gyrus
0.03	1.00	0.04	5.13	4.53	24	17	47	R	middle and superior frontal gyrus
0.03	1.00	0.05	5.07	4.48	−54	−10	−10	L	superior and middle temporal gyrus

## Group comparison

### 3 months postpartum

**Fig. 7:**
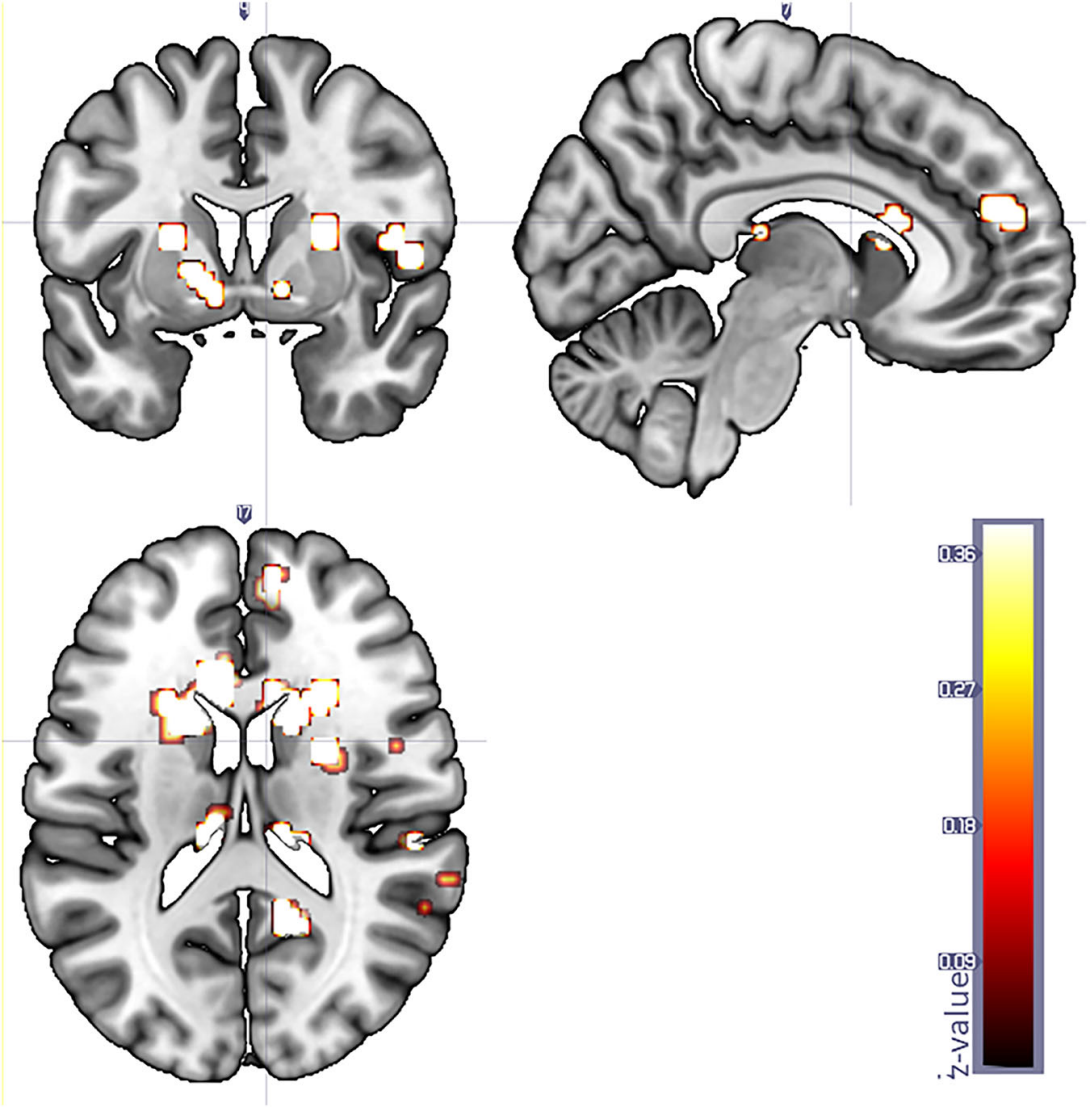
Increased functional neural activation at T1 [in BP as compared to CG] while processing [emotional baby stimuli as compared to neutral baby stimuli].

Comparing the groups at their first assessment (T1) shows a significant difference in processing emotional baby faces, irrespectively of Go or NoGo instruction and of valence. Mothers with BP showed stronger reactions in midline areas such as anterior and medial cingulate and basal ganglia of the dorsal striatum, see Fig. 7 and Table 4. No group differences or interactions reached significance for adult faces or symbols.

**Table 4:** Activations pattern for BP larger than CG in the response to emotional baby faces as compared to neutral baby faces at 3 months postpartum.

cluster	cluster size	peak	peak	peak	NMI coordinates (mm)			hemis phere	region
p(FWE-corr)	equivk	p(FWE-corr)	T	equivZ	x	y	z		
0.02	95.00	0.03	5.40	4.68	−9	20	20	L	middle and anterior cingulate
		0.32	4.44	3.99	−21	8	14	L	caudate and putamen
0.57	17.00	0.05	5.18	4.53	27	−1	14	R	putamen and anterior insula
0.03	82.00	0.47	4.24	3.85	15	11	17	R	caudate
		0.48	4.22	3.83	24	23	32	R	middle and superior frontal gyrus
		0.64	4.04	3.69	24	17	17	R	caudate

### 6 months postpartum

**Fig. 8:**
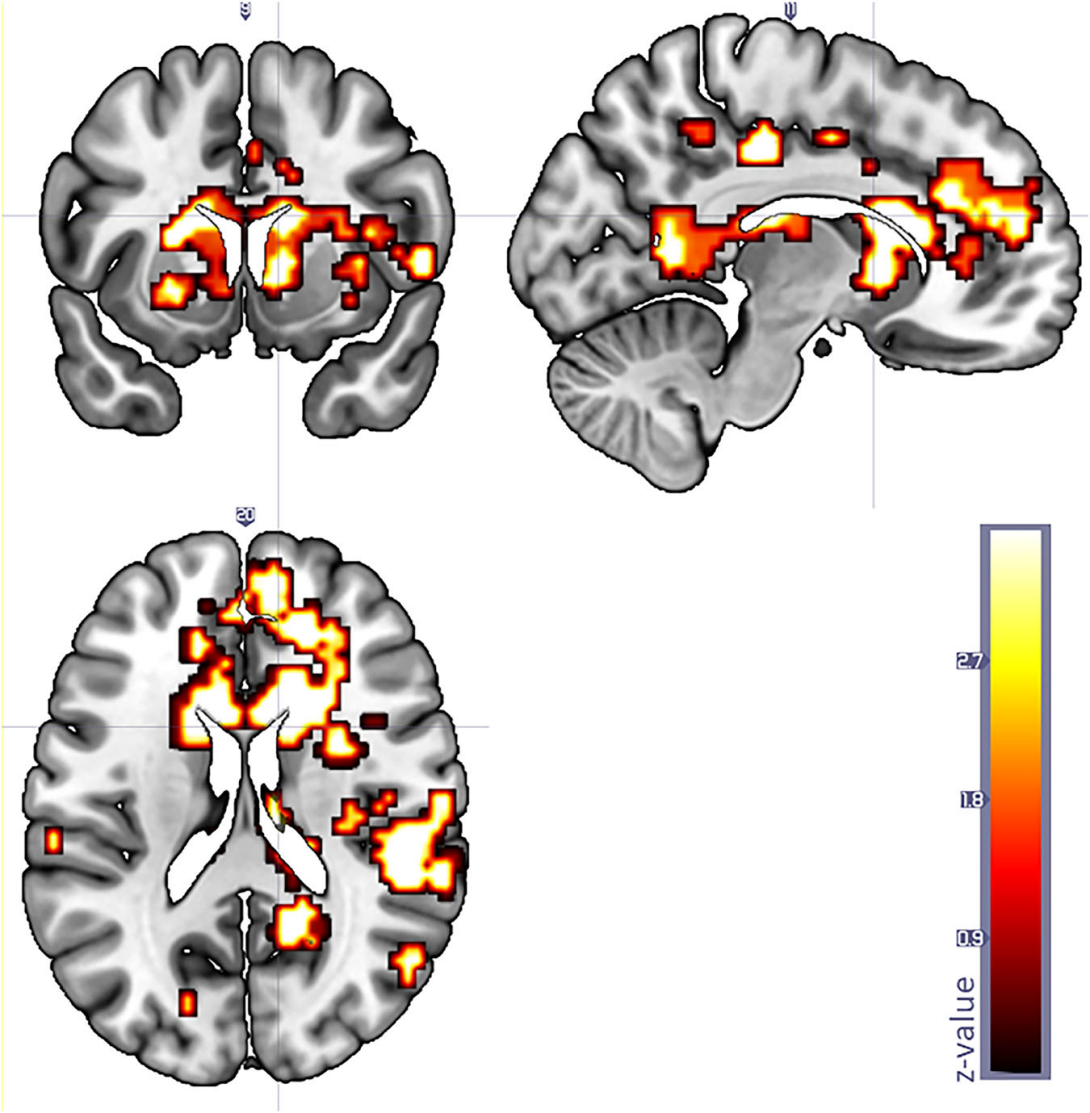
Increased functional neural activation in [BP as compared to CG] [at T2 as compared to T1] while processing [emotional baby stimuli as compared to neutral baby stimuli].

Comparing the differences of the first (T1) and second (T2) assessment between groups there is even an increase in the difference. While processing emotional baby faces, irrespectively of Go or NoGo instruction and of valence, mothers with BP increase their response in frontal gyruses, cingulate, caudate and temporal areas, see Fig. 8 and Table 5. They had received an intervention between these assessments.

**Table 5:** Stronger activation for T2 than T1 for BP larger than CG in the response to emotional baby faces as compared to neutral baby faces.

cluster	Cluster size	peak	peak	peak	MNI coorrdinates (mm)			hemis-phere	region
p(FWE-corr)	equivk	p(FWE-corr)	T	equivZ	x	y	z		
0.00	1238.00	0.00	6.32	5.27	27	29	29	R	middle and superior frontal gyrus
		0.00	6.09	5.13	12	14	17	R	caudate
		0.02	5.52	4.76	−18	14	17	L	caudate
0.00	740.00	0.02	5.49	4.74	51	−28	20	R	partietal operculum and planum temporale
		0.08	5.08	4.46	42	−58	5	R	middle temporal and inferior occipital gyrus
		0.12	4.88	4.32	60	−31	8	R	superior and middle temporal gyrus
0.00	184.00	0.12	4.90	4.33	15	−52	17	R	precuneus and posterior cingulate
		0.44	4.31	3.90	15	−31	17	R	thalamus
		0.83	3.84	3.54	9	−19	20	R	thalamus
0.13	45.00	0.18	4.72	4.21	12	−25	41	R	precentral gyrus. middle and posterior cingulate
		0.93	3.65	3.38	21	−22	41	R	precentral gyrus. middle and posterior cingulate
0.00	122.00	0.29	4.51	4.05	−24	−46	−16	L	fusiform gyrus. lingual gyrus and parahippocampal gyrus
		0.75	3.94	3.61	−24	−52	−31	L	cerebellum
		0.80	3.87	3.56	−3	−49	−16	L	cerebellum

### 12 months postpartum

**Fig. 9:**
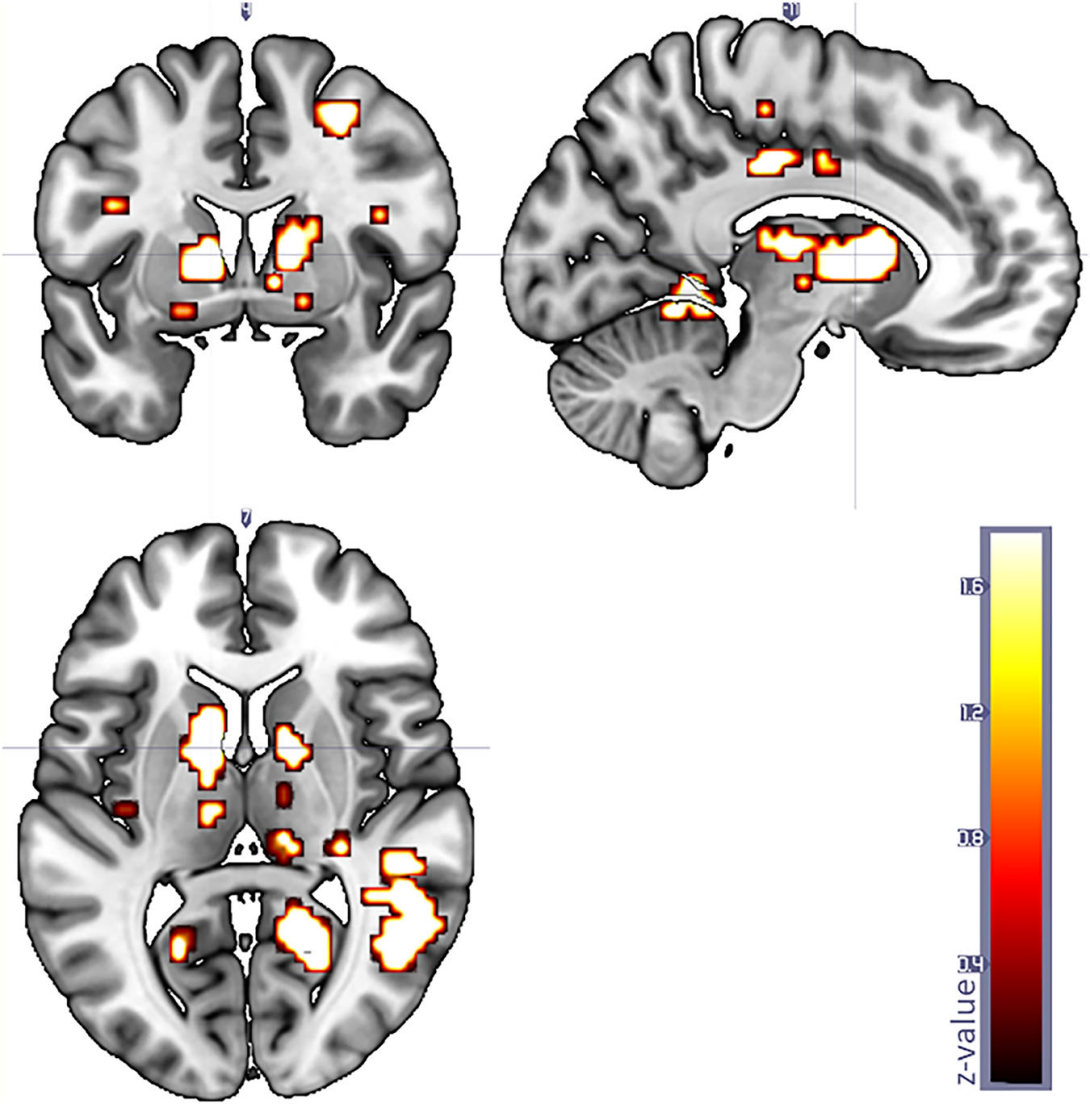
Functional neural activation stronger at for [BP than CG] at [T3 than T1], in response to [emotional baby stimuli as compared to neutral stimuli].

Comparing T3 to T1, mothers with BP still had increased activation in response to emotional baby faces as compared to controls in regions like temporal gyrus, caudate and posterior cingulate, yet this difference was not as large as at T2, see Fig. 9 and Table 6.

**Table 6:** Stronger activation for T3 than T1 for BP larger than CG in the response to emotional baby faces as compared to neutral baby faces.

cluster	cluster	peak	peak	peak					
p(FWE-corr)	equivk	p(FWE-corr)	T	equivZ	x.y.z mm	x.y.z mm	x.y.z mm		
0.00	340.00	0.09	4.98	4.39	57	−46	14	R	middle and superior temporal gyrus and angular gyrus
		0.14	4.80	4.26	63	−37	14	R	superior and middle temporal gyrus and supramarginal gyrus
		0.34	4.40	3.97	48	−43	23	R	angular, superior temporal and supramarginal gyurs
0.34	28.00	0.11	4.88	4.32	42	−40	−13	R	fusiform and inferior temporal gyrus
0.03	82.00	0.24	4.56	4.08	−12	8	8	L	caudate and putamen
		0.40	4.32	3.91	−12	−1	5	L	thalamus. pallidum and caudate
0.00	179.00	0.29	4.47	4.02	24	−61	8	R	calcarine cortex. precuneus and lingual gyrus
		0.42	4.29	3.89	12	−52	−1	R	lingual gyrus and posterior cingulate
		0.67	4.00	3.66	15	−52	14	R	precuneus and posterior cingulate gyrus

No significant group effects for Go vs. NoGo or the valence of the facial expression was found. Using EPDS as covariate did not result in other or EPDS-related effects for any of the comparisons.

Additional exploratory correlational analyses between neural activity, task performance and self-reported bonding are reported in the supplemental materials.

## Discussion

In this report, we present and validate an adapted version of the GoNoGo Task including infant stimuli in a sample of mothers with bonding problems (BP) towards their infant. The Infant Emotional GoNoGo Task tests specifically for the perception of and reaction to infant faces with positive, negative, and neutral valence. Our general results suggest that inhibition (NoGo condition) to infant faces activates a broad network for emotion processing both in mothers with and without bonding disorder, including the frontal gyrus, putamen, insula and cingulate cortex. In contrast to the adult faces condition, this activity was even stronger in superior frontal gyrus, nucleus caudatus, accumbens and putamen during the infant faces. This neural network is in line with similar studies using infant stimuli (e.g. [32]), and indicates a high salience of emotional infant faces in particular in mothers in the postpartum period. On a behavioral main effect level, mothers showed significantly higher error rates of inhibition in the emotional nogo condition as well as slower reactions in the emotional go condition for baby faces compared to adult faces, indicating a higher effort in emotional inhibition for baby faces.

At T1 there were no group differences between mothers with BP and healthy controls in reaction times or error rates of inhibition. Interestingly, the exploratory analysis on inhibition error rates across all three time points, revealed lower inhibition error rates in mothers with BP compared to healthy controls at T2, suggesting an experience-based or intervention-based (neurofeedback intervention between T1 and T2) improvement of emotion processing in mothers with BP during the first months with their child.

Both the processing and reaction to emotional stimuli are however relevant precursors of broader emotion regulation capacities [63, 64]. Challenges in emotion regulation in general and specifically the perception of positive affect in others are reported for clinical populations, but the specific behavioral patterns have not yet been shown for BP).

On a neural level, a difference between groups at T1 (3 months postpartum) shows that neural response networks comprising insula, cingulate cortex and caudate are stronger activated towards emotional infant stimuli in BP than in CG. While the anterior insular cortex is prominently known for its role in social emotions, especially in anticipating and learning from others’ mental states, the ACC is considered a central node of the emotional processing and regulation network to control affective states on a limbic system level and the caudate as part of the dorsal striatum is considered part of the dopaminergic motivational network. All of them are not only functionally connected but also the network has been discussed to be altered in psychopathology. In addition, they are also involved in the parental brain network that is supposed to be strengthened after becoming a mother while depressed mothers show hypoactivation in the associated brain regions [36–38]. The evidence of stronger activations of this network could therefore be a correlate of higher effort and usage of neural resources in order to cope with the affective content. This is in line with reports of heightened ACC and caudate activation especially for processing stimuli of a distressed child both in healthy and clinical individuals [65, 66]. Our finding is in contrast to the hypoactivation reported for mothers with postpartum depression, thus indicating different mechanisms underlying postpartum depression and BP.

Additional exploratory analyses have found that towards 6 months postpartum, in line with the behavioral improvement in mothers with BP at T2, the difference between BP and CG in brain activity has even increased in the aforementioned areas, possibly following the neurofeedback intervention between 3 and 6 months and therefore to be interpreted with caution. Towards 12 months postpartum, the neural activation patterns of the mothers with BP came again closer to the neural pattern of the CG, leaving still group differences in e.g. cingulate and caudate, yet less pronounced than at 6 months. Together with the behavioral adjustment, this might be an effect of compensation and experience-based improvement in infant emotion processing. Either the neurofeedback intervention or the intense first months of motherhood may have stimulated the usage of their neural systems [19] - yet no clear conclusion can be drawn based on our study design. It has been a more general observation for persons with psychological disorders to need increased brain activity for some disorder-specific tasks (for instance for self-referential processing in social anxiety, or working memory in obsessive-compulsive disorder). As no intervention took place between 6 and 12 months, this might be interpreted as a normalization of neural systems and a mostly successful adaptation to the new situation (see other publications from our team).

Interestingly, in all behavioural and neural analyses, depressive symptoms did not result in any significant or relevant effects, although the sample had subclinical symptoms (that were close to the cut-off score of the EPDS). This adds to the assumption that bonding disorder is probably distinct from postpartum depression and needs to be considered as own specific clinical field [48].

Surprisingly and contrary to expectations based on the state of research, we did not observe any effects of stimulus valence, namely positive or negative affect of the infant face. This could be due to several reasons. One likely reason is that power issues prevented detection of an effect as the sensitivity analysis suggests that our sample was too small to show small effect sizes. It is also possible that there is impairment towards both positive and negative valence, though: Infant cues are highly salient stimuli that activate the caregiving system largely independent of their specific emotional expression. Especially in the early postpartum period, neural responses may primarily reflect the relevance of “infant” versus “non-infant” stimuli rather than fine-grained affective valence. Activation in quite unspecific regions such as the ACC likely indexes salience detection and caregiving-related motivation rather than valence coding per se. Consistent with this view, emotional infant faces elicited stronger responses than adult faces across all mothers. Clinically, bonding problems may therefore not be characterized by specifically heightened reactivity, late responses or errors towards negative infant cues. Instead, they may reflect altered motivational-affective relevance processing.

Taken together we are able to show that neural activation associated with emotion processing of baby stimuli is stronger in mothers with BP as compared to healthy controls 3 months postpartum.

Our study has some limitations that need to be taken into account for the interpretation of the data and for the planning of future studies. First, and most importantly, all mothers with BP participated in an individualized fMRI neurofeedback between T1 and T2 that was associated with improved clinical symptoms. This overall improvement was not associated with specific task performance or neural activation to the GoNoGo-task, however it should be interpreted as a potential confounder with the results at 6 and 12 months postpartum. Another limitation that might hamper generalization of results is the sample characteristics and size. Although aiming for bonding issues as inclusion criterion, there are higher levels of subclinical depression in this group, which results in multicollinearity problems during statistical analyses. Therefore, we cannot absolutely rule out that group differences are also related to depressiveness. Also, as a criterion for participation, the mothers were burdened on a medium level: Severe cases had to be forwarded to immediate therapeutic treatment, while unclear and very mild reports of problems did not lead to inclusion in the BP group. The broad range of clinical comorbidity, however, did not apparently reduce the effect and might be argued rather as strength of the study and for the robustness of findings. Lastly, the generalization of results is clearly limited to women. Future studies might include fathers and other attachment persons. In addition, assessing emotion processing and brain networks already before birth or even pregnancy might help to understand the specific parental brain adaptations and their causes better, both for healthy and clinical individuals.

Our results have some broader clinical implications as well. Our data indicates deficits of the neural emotion processing network that are specific for infant stimuli and for mothers with bonding problems, that might be under the influence of learning/experience-based adaptations during the 1st year postpartum, irrespective of depressive symptoms. This adds to the assumption that BP should not be treated as equivalent to depressiveness, but has distinct features - as for instance in emotion processing and neural activation in the associated brain regions.

Moreover, our findings underscore the potential value of targeting parental responses to infant affect as a focus of early preventive and therapeutic strategies. Interventions that enhance parental sensitivity to infant cues and support adaptive regulation of infant-related emotional responses may be particularly relevant during the early postpartum period, even in populations not meeting criteria for a depressive disorder.

## Supplementary information

Suppl methods and results

## Data Availability

Eckstein, Monika, 2026, Improving Maternal Bonding Through Real-time fMRI Neurofeedback in Mothers with Postpartum Bonding Problems [data], 10.11588/DATA/IA4G1O, heiDATA, V1, UNF:6:49QHbDmHzKMHCHrSiZ1IYg== [fi leUNF].
