# Supplementary material for: Postpartum maternal bonding problems relate to aberrant neural processing of infant emotions: Results of an adapted fMRI emotional GoNoGo task
Source: Transl Psychiatry. 2026 Jul 6;16:343. doi: 10.1038/s41398-026-04231-y (PMC13341878; doi:10.1038/s41398-026-04231-y)
Supplement: Supplementary file 1 — Suppl methods and results [file 41398_2026_4231_MOESM1_ESM.docx]

**Supplemental methods and results**

Postpartum maternal bonding problems relate to aberrant neural processing of infant emotions: Results of an adapted fMRI Emotional GoNoGo Task

Monika Eckstein, Marlene Krauch, Ines Brenner, Beate Ditzen, Anna-Lena Zietlow

**Methods**

**fMRI task: Infant Emotional GoNoGo Task**

Using a specifically adapted GoNoGo paradigm, participants were presented with positive, negative and neutral expressions of pictures of unknown babies, aged approx. 4-10 months, and, unknown adults with positive, negative and neutral expressions, as well as non-social control stimuli (geometric figures; a circle, a cross, a diamond and a triangle) over six presentation blocks. Facial stimuli were taken from an established database (57) with positive, neutral and negative affect in infants with age 3-5 months and the Karolinska faces for adults, see Figure 2.

The following factors were systematically manipulated: child versus adult and emotionality of facial expression (positive vs negative vs. neutral). In two blocks, the participants received instructions to respond by pressing a button as fast as possible (Go trials) to all facial expressions except (NoGo trials) the negative (one block babies, one block adults). In two other blocks, they were instructed to respond as fast as possible to all except the positive stimuli (one block babies, one block adults). In the two non-social blocks, the participants were instructed to react as fast as possible to all shapes but not to a circle or a diamond.

Each block consisted of 12 pictures shown twice for 500ms, therefore 24 trials, of which 8 were NoGo trials. Fixation cross between trials was jittered from 1500-2000ms. Between blocks was an interval of 5000ms. Total task duration was approx. 14 minutes.

**fMRI data acquisition**

Imaging was performed using a 3Tesla Prisma-Fit Siemens Scanner (Siemens, Erlangen, Germany) at the Department of Neuroradiology at University Hospital Heidelberg. First a detailed anatomical scan was obtained with a magnetization prepared rapid gradient echo (MPRAGE) sequence with repetition time TR=1.9s, echo time TE=2.52ms, flip angle=9° and an isotropic resolution of 1x1x1mm, followed by functional scans. Functional images were acquired with an Echo Planar Imaging (EPI) sequence with TR=1.64s, TE=30ms, flip angle=73° and GRAPPA factor 2 in 30 slices of 3mm thickness, and field of view FoV=192mm for a voxel size of 3x3x3mm.

**Analyses**

Behavioral data were analysed using IBM SPSS Statistics (version 31). We focused on reaction times (go trials) and on error rates of inhibition (nogo trials). Data for T1 were analysed using paired-samples t-tests and MANOVAs for between-group comparisons. In an exploratory approach, repeated-measures ANOVAs were conducted to compare reaction times and error rates across T1, T2 and T3.

Outliers were excluded using a window of 100ms-1500ms as a range for plausible values. Blocks with >60% implausible values or response errors were interpreted as failure to understand the task instruction. This resulted in 21-40% subsequent dismissal from analyses, see Supplementary Table S1.

Supplementary Table S1: Excluded data for reaction times and error rates

|  | Missings due to missing of experimental session | Blockwise exclusion due to invalide or unplausible values |
| --- | --- | --- |
| T1 | 0 (0%) | 26 (40%) |
| T2 | 9 (13.8%) | 21 (32.3%) |
| T3 | 17 (26.2%) | 14 (21.5%) |

For fMRI data, using SPM12 (Wellcome Center for Human Neuroimaging, London, UK) we first conducted preprocessing of the functional data with the following steps: Slice time correction, realignment to the first image. Anatomical images were segmented and normalized to the SPM 12 NMI template. Functional images were coregistered with these anatomical images and normalized in NMI space rescaling to voxel size 2x2x2mm. After smoothing with a full width at half maximum FWHM=8x8x8mm Gaussian kernel. The first 5 images of each session were discarded.

On the first level, we specified an event-related model with 3 sessions and for each the 12 task conditions as saved in the logfiles with the onsets of symbols, adult positive faces, adult negative faces, baby positive faces and baby negative faces, both as Go and NoGo conditions, and neutral adult and neutral baby faces as Go condition. Interstimulus intervals entered the implicit baseline. We controlled for the movement regressors obtained from preprocessing and applied a high pass filter of 128 and convolved the conditions with the canonical hemodynamic response function. The estimated model calculated contrasts for the relevant conditions such as T1[emotional baby face > neutral baby face] and Mean [[baby face NoGo > baby face Go] > [adult face NoGo > adult face Go]]. On the second level, participant-specific contrast maps from the first level analyses were compared between patients and controls with two-sample t-tests and EPDS scores were tested as covariates. A significance threshold of p=0.05 cluster-level FWE-corrected with cluster-defining height threshold of p=0.001 uncorrected was applied. Probabilistic labelling of regions for the tables was done using SPM built-in Neuromorphometrics atlas.

Correlational analyses including neural data used parameter estimates extracted using MarsBar toolbox (Brett et al, 2002) for anatomical defined ROIs of the ACC and Ncl. Caudate (left and right separately) exported from AAL atlas (Tzourio-Mazoyer et al, 2002).

**Exploratory results**

To explore whether task behaviour and neural response during the task are related to bonding problems, correlational analyses were performed over all participants As this resulted a large number of correlations, we will present all results for T1 in supplementary Table S2 and supplementary Figures S1-S5. P-values are reported uncorrected and significance has to be treated with high caution given probably alpha-error inflation.


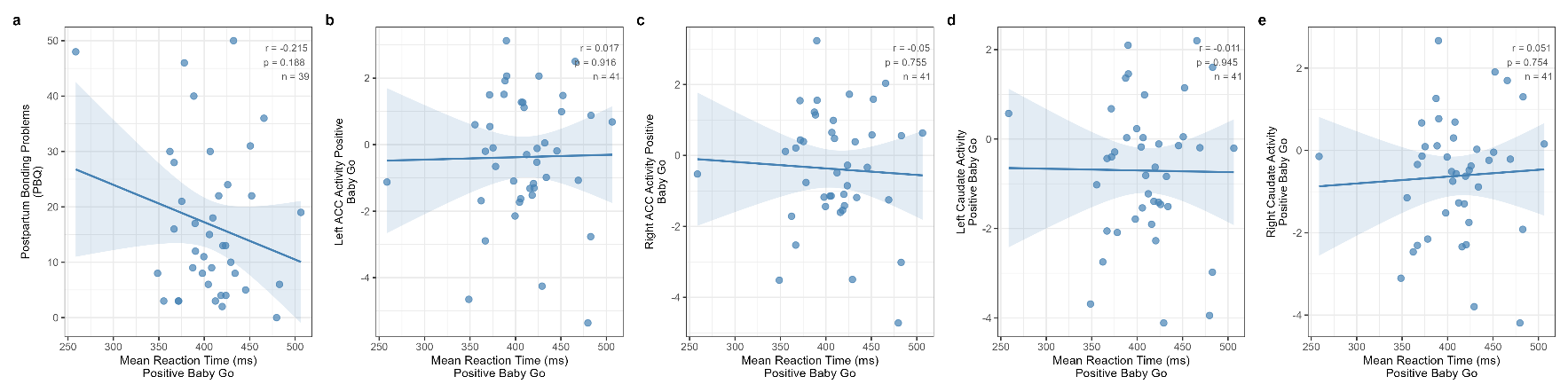


Supplementary Figure S1: Correlations between reaction time to positive baby faces and postpartum bonding problems or neural activation during Go trials. PBQ (panel a) or ACC and Caudate parameter estimates for positive baby Go trials (panels b–e). Lines represent linear regression fits; shading indicates 95% confidence intervals. r = Pearson correlation coefficient; † p < .10; * p < .05; ** p < .01; *** p < .001.


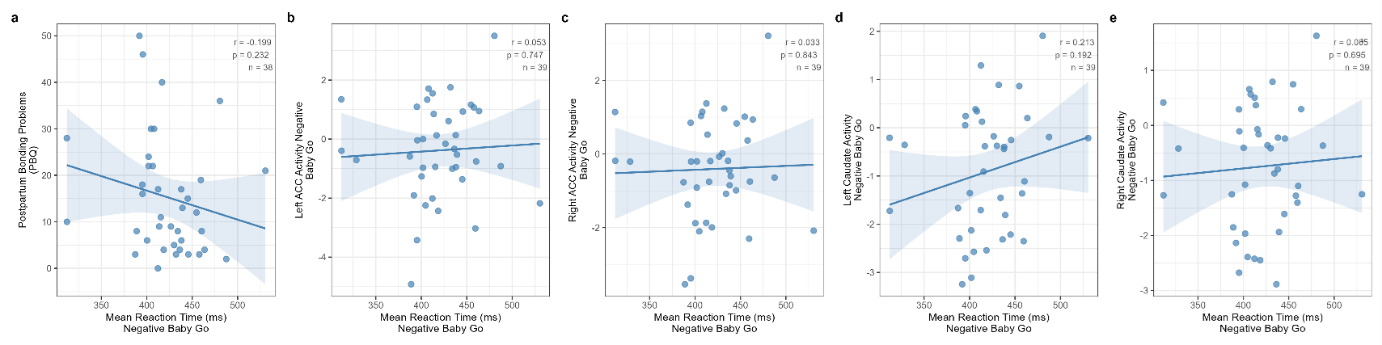
Supplementary Figure S2: Correlations between reaction time to negative baby faces and postpartum bonding problems or neural activation during Go trials. PBQ (panel a) or ACC and Caudate parameter estimates for negative baby Go trials (panels b–e). Lines represent linear regression fits; shading indicates 95% confidence intervals. r = Pearson correlation coefficient; † p < .10; * p < .05; ** p < .01; *** p < .001.


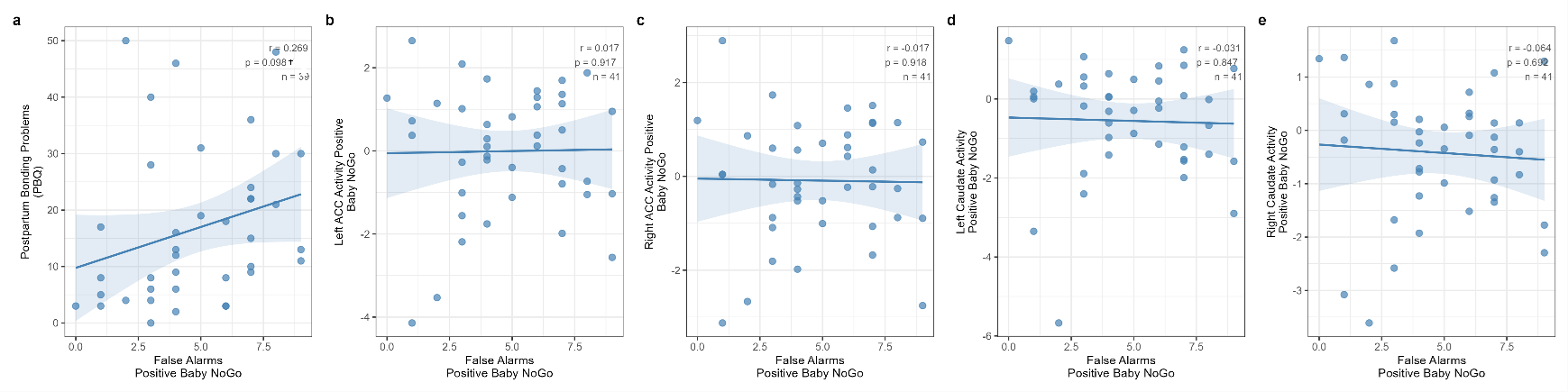


Supplementary Figure S3: Correlations between false alarm rate to positive baby faces and postpartum bonding problems or neural activation during NoGo trials. Scatterplots show Pearson correlations between false alarm rates to positive baby stimuli and postpartum bonding problems (PBQ; panel a) or bilateral ACC and Caudate parameter estimates for positive baby NoGo trials (panels b–e). Lines represent linear regression fits; shading indicates 95% confidence intervals. r = Pearson correlation coefficient; † p < .10; * p < .05; ** p < .01; *** p < .001.


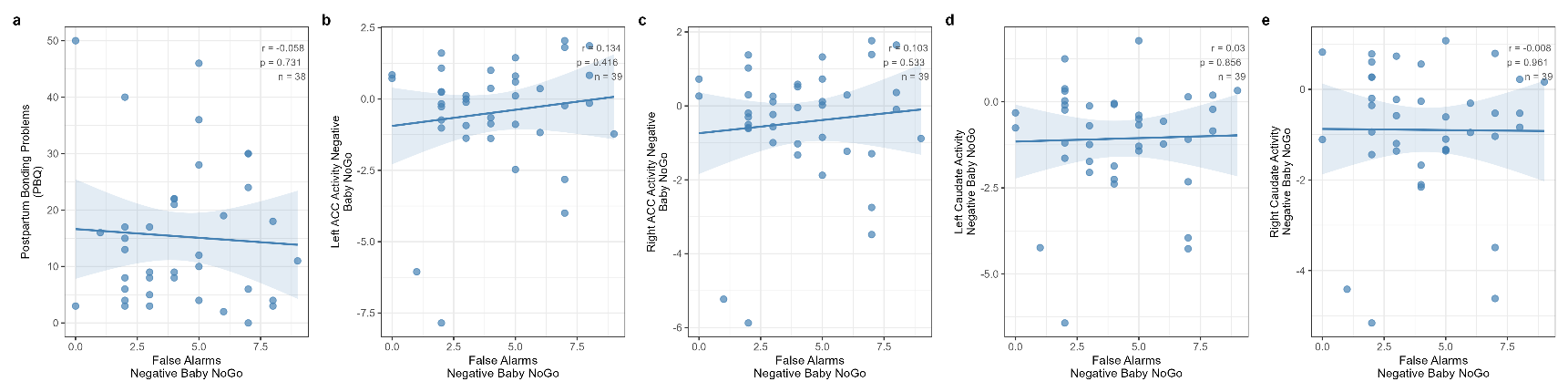


Supplementary Figure S4: Correlations between false alarm rate to negative baby faces and postpartum bonding problems or neural activation during NoGo trials. As Figure S3, for negative baby stimuli and corresponding negative baby NoGo neural activation.


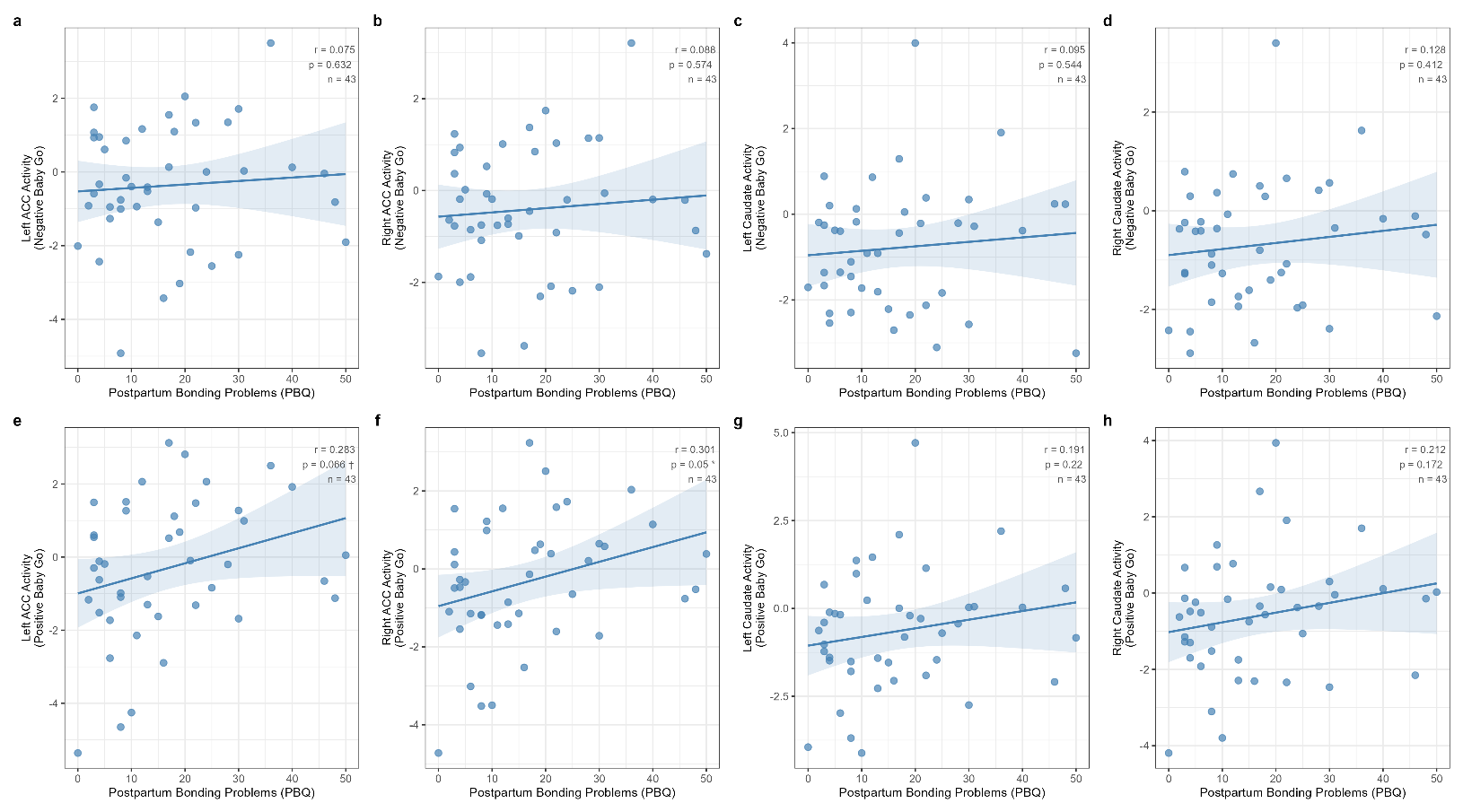


Supplementary Figure S5: Correlations between postpartum bonding problems and neural activation during Go trials. Scatterplots show Pearson correlations between PBQ scores and ACC and Caudate parameter estimates for negative baby (panels a–d) and positive baby Go trials (panels e–h). Lines represent linear regression fits; shading indicates 95% confidence intervals. r = Pearson correlation coefficient; † p < .10; * p < .05; ** p < .01; *** p < .001.

Correlations with change scores between T1, T2 and T3 as well as correlations at T2 and T3 did not result in any relevant correlation coefficients.

Supplementary Table 2: Correlational analyses at T1

|  |  | **n** | **Pearson’s correlation coefficient** | **p-value** |
| --- | --- | --- | --- | --- |
| Reaction time towards positive baby face (Go) | Postpartum bonding problems (PBQ Score) | 39 | -0.215 | 0.1883 |
|  | Parameter estimates for left ACC activity | 41 | 0.017 | 0.9162 |
|  | Parameter estimates for right ACC activity | 41 | -0.050 | 0.7553 |
|  | Parameter estimates for left caudate activity | 41 | -0.011 | 0.9452 |
|  | Parameter estimates for right caudate activity | 41 | 0.051 | 0.7537 |
| Reaction time towards negative baby face (Go) | Postpartum bonding problems (PBQ Score) | 38 | -0.199 | 0.2316 |
|  | Parameter estimates for left ACC activity | 39 | 0.053 | 0.7467 |
|  | Parameter estimates for right ACC activity | 39 | 0.033 | 0.8426 |
|  | Parameter estimates for left caudate activity | 39 | 0.213 | 0.1920 |
|  | Parameter estimates for right caudate activity | 39 | 0.065 | 0.6949 |
| Error rates towards negative baby face | Postpartum bonding problems (PBQ Score) | 38 | -0.058 | 0.7313 |
|  | Parameter estimates for left ACC activity | 39 | 0.134 | 0.4161 |
|  | Parameter estimates for right ACC activity | 39 | 0.103 | 0.5328 |
|  | Parameter estimates for left caudate activity | 39 | 0.030 | 0.8559 |
|  | Parameter estimates for right caudate activity | 39 | -0.008 | 0.9612 |
| Error rates towards positive baby face | Postpartum bonding problems (PBQ Score) | 39 | 0.269 | 0.0982 |
|  | Parameter estimates for left ACC activity | 41 | 0.017 | 0.9171 |
|  | Parameter estimates for right ACC activity | 41 | -0.017 | 0.9181 |
|  | Parameter estimates for left caudate activity | 41 | -0.031 | 0.8474 |
|  | Parameter estimates for right caudate activity | 41 | -0.064 | 0.6916 |
| Postpartum bonding problems (PBQ Score) | Parameter estimates for left ACC activity towards negative baby | 43 | 0.075 | 0.6317 |
|  | Parameter estimates for right ACC activity towards negative baby | 43 | 0.088 | 0.5737 |
|  | Parameter estimates for left caudate activity towards negative baby | 43 | 0.095 | 0.5439 |
|  | Parameter estimates for right caudate activity towards negative baby | 43 | 0.128 | 0.4119 |
|  | Parameter estimates for left ACC activity towards positive baby | 43 | 0.283 | 0.0660 |
|  | Parameter estimates for right ACC activity towards positive baby | 43 | 0.301 | 0.0497 |
|  | Parameter estimates for left caudate activity towards positive baby | 43 | 0.191 | 0.2203 |
|  | Parameter estimates for right caudate activity towards positive baby | 43 | 0.212 | 0.1717 |

*Note: P-values are reported uncorrected. Reaction-time measures are labelled by the Go-stimulus they were measured on (consistent with Supplementary Figures S1–S2); neural parameter estimates are paired with the matching baby-emotion condition.*

To explore associations between depression symptoms and bonding problems, we calculated a correlation between PBQ scores and EPDS scores at T1 for all available N = 62 questionnaires which resulted in r= 0.585 p < 0.001, see Supplementary Figure S6. Separate correlations r =0.177, p = 0.317 in the intervention group and r = 0.323, p = 0.093 for healthy participants, therefore the correlation is mainly driven by subclinical persons.


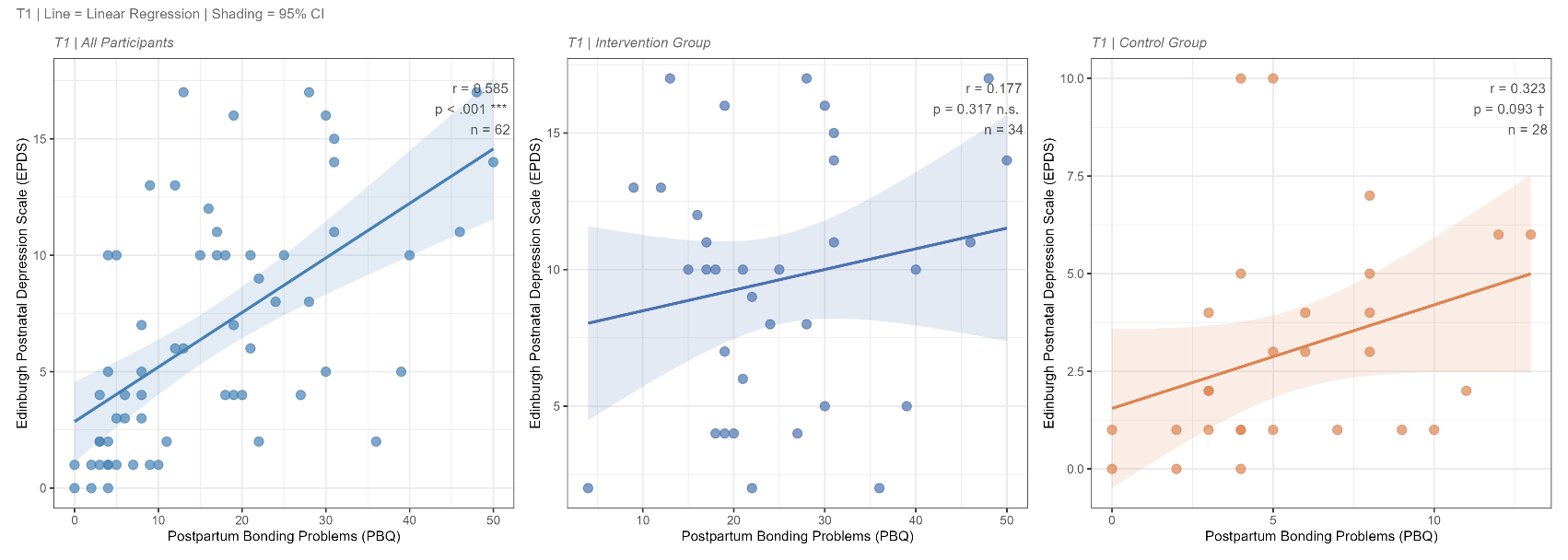


Supplementary Figure S6: Correlation between depressiveness (EPDS) and bonding problems (PBQ) over all participants (a) and separately for groups (b Intervention group, c control group). Line indicates linear regression; shading indicates 95% CI.

**References**

Brett, M., Anton, J. L., Valabregue, R., & Poline, J. B. (2002). Region of interest analysis using the MarsBar toolbox for SPM 99. *Neuroimage*, *16*(2), S497.

Tzourio-Mazoyer, N., Landeau, B., Papathanassiou, D., Crivello, F., Etard, O., Delcroix, N., ... & Joliot, M. (2002). Automated anatomical labeling of activations in SPM using a macroscopic anatomical parcellation of the MNI MRI single-subject brain. *Neuroimage*, *15*(1), 273-289.
